# Extracellular Vesicle-embedded alginate hydrogel patch for accelerated wound healing

**DOI:** 10.1016/j.mtbio.2026.103288

**Published:** 2026-05-27

**Authors:** Won Ho Jang, Van Dat Bui, Van Hieu Duong, Sol Shin, Jungmi Lee, Torsha Ghosh, Chang Hyun Lee, Soyoung Son, Jiyeon Kim, Eun-Cheol Lee, Suk Ho Bhang, Jae Hyung Park

**Affiliations:** a School of Chemical Engineering, College of Engineering, Sungkyunkwan University (SKKU), Suwon, 16419, Republic of Korea; b Vinmec Research Institute of Stem Cell and Gene Technology, College of Health Science, VinUniversity, Vinhomes Ocean Park, Gia Lam District, Hanoi, 1310, Viet Nam; c Department of Integrative Biological Sciences and Industry, Sejong University, Seoul, 05006, Republic of Korea; d Department of MetaBioHealth, School of Medicine, Sungkyunkwan University (SKKU), Suwon, 16419, Republic of Korea; e Department of Health Sciences and Technology, SAIHST, Sungkyunkwan University, Suwon, 16419, Republic of Korea; f Biomedical Institute for Convergence at SKKU (BICS), Sungkyunkwan University, Suwon, 16419, Republic of Korea

**Keywords:** Extracellular vesicles, Wound healing, Epigallocatechin gallate, Hydrogel, Anti-inflammation, Controlled release

## Abstract

The process of skin wound healing is markedly impaired by elevated levels of reactive oxygen species (ROS) and the persistent presence of pro-inflammatory macrophages in the injured tissue. To address these pathological challenges, we herein developed an alginate-based hydrogel patch (EGEV-Gel) engineered for the sustained release of human adipose stem cell-derived extracellular vesicles (hASC-EVs) and epigallocatechin gallate (EGCG). hASC-EVs were incorporated to promote tissue regeneration, while EGCG served as a potent ROS scavenger. In an *in vitro* wound healing model using the transwell insert system, EGEV-Gel demonstrated remarkable efficacy in suppressing M1 macrophage polarization, scavenging excessive ROS, and restoring the functional capacity of dermal fibroblasts and endothelial cells compromised by oxidative stress. When applied to the acute wound-induced mouse, EGEV-Gel significantly reduced the M1 macrophage population and the ROS level at 3 days post-treatment. Histological analysis of the skin layers further confirmed restoration of skin architecture, as demonstrated by the normalized thickness of key structural components, including the epidermis, collagen layer, and granulation tissue. These findings highlight the therapeutic potential of the hASC-EVs/EGCG-loaded hydrogel patch as a promising platform for promoting wound repair and modulating the inflammatory microenvironment in cutaneous injuries.

## Highlights

• Wound healing is delayed by reactive oxygen species (ROS) and pro-inflammatory cells.
• Hydrogel releases extracellular vesicles (EVs) and epigallocatechin gallate (EGCG).
• EGCG acts as a potent ROS scavenger for anti-inflammation.
• EVs restore ROS-damaged cellular functions and promote tissue regeneration.
• Synergistic effects suppress inflammation and accelerate functional wound healing.

## Graphical abstract

**Figure:**
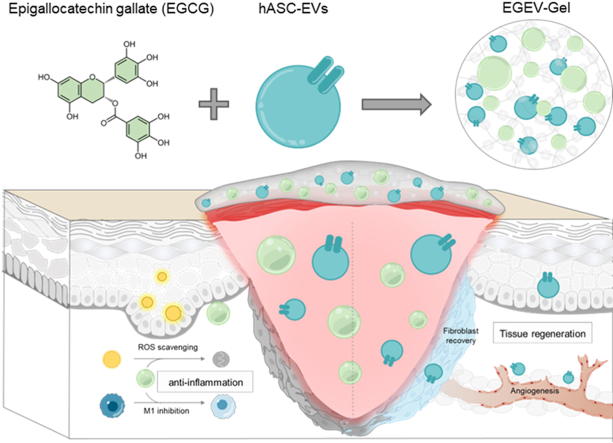

## 1 Introduction

Cutaneous wounds and impaired healing processes represent significant public health challenges [1]. In particular, large wounds resulting from trauma, acute illness, or major surgical procedures often require several weeks to heal and commonly result in fibrotic scarring, which can compromise tissue function [2]. Therefore, the development of therapeutics that not only accelerate the healing process but also minimize scar formation is urgently needed. It is well established that damaged cells and pro-inflammatory M1 macrophages release reactive oxygen species (ROS), which play a crucial role in recruiting inflammatory cells to the wound site [2]. However, excessive levels of M1 macrophages and ROS can disrupt the healing cascade, delay tissue repair, and contribute to persistent scarring. Thus, strategies aimed at reducing M1 macrophage activity and mitigating ROS accumulation are considered beneficial for promoting comprehensive skin wound regeneration.

Mesenchymal stem cell-derived extracellular vesicles (MSC-EVs) have emerged as a potent class of biologics for skin wound healing, owing to their favorable safety profile and therapeutic efficacy [3]. MSC-EVs, enriched with a variety of growth factors, microRNAs, and cytokines, possess intrinsic anti-inflammatory and regenerative properties that contribute to the repair of complex skin structures, including the epidermis, dermis, hair follicles, and blood vessels [3]. By enhancing cellular proliferation, reducing apoptosis, and modulating oxidative stress, MSC-EVs support the recovery of damaged tissue and promote overall wound regeneration [[4], [5], [6]]. In addition, they suppress M1 macrophage polarization by regulating multiple inflammatory signaling pathways, thereby reducing the production of pro-inflammatory cytokines [[7], [8], [9], [10]]. Among them, hASC-EVs were selected due to their high translational feasibility as an autologous and scalable platform [11], as well as their comparable capacity to modulate oxidative stress and macrophage polarization relative to other MSC-derived EVs [12,13]. Despite these advantages, the clinical application of MSC-EVs has been limited by their rapid clearance from the wound site. Therefore, sustained delivery systems are essential to maintain their bioactivity for a long period of time and maximize their therapeutic effect in wound repair.

Alginate, a natural polysaccharide, has been widely explored as a versatile biomaterial in wound healing applications [14]. Its structure consists of blocks of β-D-mannuronic acid and α-L-guluronic acid residues, which can undergo ionic crosslinking in the presence of divalent cations such as calcium ions, forming alginate-calcium hydrogels [15]. These hydrogels are particularly attractive for biomedical applications due to their biocompatibility, rapid gelation under physiological conditions, and tunable mechanical properties [16]. Furthermore, their porous structure allow for the encapsulation and controlled release of therapeutic agents, making them ideal candidates for delivering bioactive molecules to wound sites [17]. When applied as a dried hydrogel formulation, the alginate hydrogel patch can absorb wound exudate, thereby triggering the release of therapeutic agents at the wound site. However, most existing wound materials, including some alginate-based hydrogels limited in single-pathway modulation [18,19], as well as inorganic systems, despite enabling multimodal effects [[20], [21], [22]], remain constrained by long-term biosafety concerns, particularly in inorganic systems, due to non-biodegradable accumulation, uncontrolled ROS-mediated cytotoxicity, and limited intrinsic biocompatibility with host tissues, thereby limiting their comprehensive translational healing applications.

**Fig. 1:**
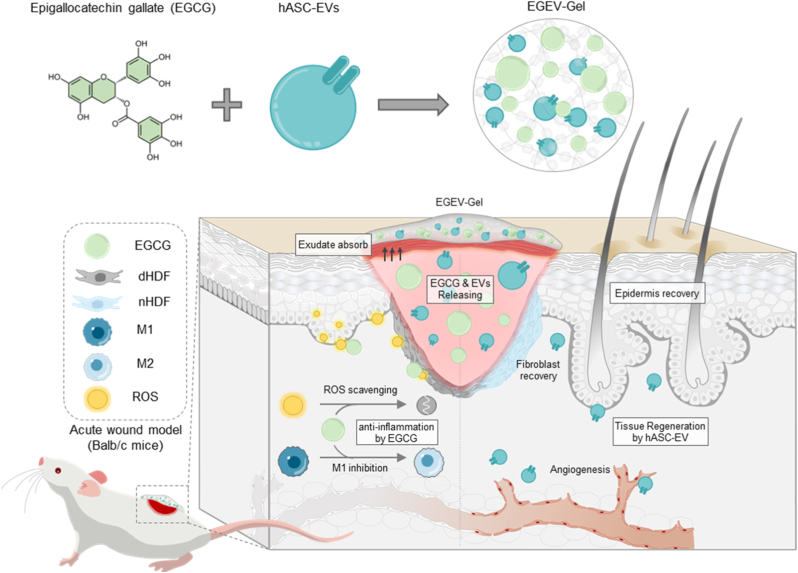
**Illustration of using alginate-based hydrogel for wound healing.** The hydrogel patch scavenges ROS and inhibits M1 macrophages, thereby accelerating wound closure while also promoting angiogenesis and hair follicle regeneration.

Given the favorable properties of alginate, we herein developed an alginate-based hydrogel patch for cutaneous wound healing, referred to as EGEV-Gel. This hydrogel patch is engineered to co-deliver hASC-EVs, along with epigallocatechin gallate (EGCG), a bioactive polyphenol known for its potent anti-inflammatory and antioxidant properties (Fig. 1). Alginate was selected as the base material for its biocompatibility, biodegradability, and ability to form hydrogels via calcium-mediated ionic crosslinking under mild, non-toxic conditions, avoiding complex chemical reactions required by other polymer systems and thereby preserving the bioactivity of sensitive therapeutics such as hASC-EVs. Furthermore, its tunable mechanical strength and high-water retention capacity make it particularly well-suited for maintaining a moist and supportive environment conducive to skin regeneration. The porous architecture of the alginate matrix allows for efficient encapsulation and sustained release of both hASC-EVs and EGCG, aiming to synergistically promote tissue regeneration while modulating the inflammatory and oxidative microenvironment of the wound. After characterizing the physicochemical properties of EGEV-Gel, we evaluated its *in vitro* effects on M1 macrophage polarization, ROS scavenging, fibroblast recovery, and angiogenesis. Finally, the therapeutic potential of EGEV-Gel was assessed *in vivo* using a mouse model of acute skin injury.

## 2 Materials and methods

### 2.1 Materials

Sodium alginate was purchased from Sigma-Aldrich (St. Louis, MO, USA). It is a linear copolymer of β-D-mannuronate (M) and α-L-guluronate (G) residues linked by (1 → 4) glycosidic bonds, with an estimated M/G ratio of ∼1.56 (61% M and 39% G). The molecular weight range is 12,000 - 80,000 Da, estimated from viscosity (15–25 cP, 1% aqueous solution). These values were provided by the supplier and are not batch-specific. Calcium chloride, and 2,2-diphenyl-1-picrylhydrazyl hydrate (DPPH) were purchased from Sigma-Aldrich (St. Louis, MO, USA). Deionized water (DIW) was purified using an AquaMax-Ultra water purification system (Younglin Co., Anyang, Korea). All other chemicals were of analytical grade and used without further purification. Fetal bovine serum (FBS), Dulbecco's modified Eagle's medium (DMEM), and Roswell Park Memorial Institute 1640 Medium (RPMI 1640) medium were purchased from Hyclone Laboratories Inc. (Logan, UT, USA). The antibiotic-antimycotic solution, phosphate-buffered saline (PBS), and trypsin-EDTA were obtained from WelGENE (Gyeongsan, Korea). 3,3′-Dioctadecyloxacarbocyanine perchlorate was obtained from Thermo Fisher Scientific (Rockford, IL, USA).

### 2.2 Cell culture

Primary human adipose stem cells (age 38, female, 70E21-062) were purchased from Cefobio Inc. (Seoul, Korea). Primary human dermal fibroblasts (HDF) and RAW264.7 cells were obtained from the American Type Culture Collection (Manassas, VA, USA). HDFs were cultured in DMEM containing 10% FBS and 1% antibiotic-antimycotic. Primary human umbilical vein endothelial cells (HUVEC) and Endothelial Cell Growth Medium MV (EGM) were obtained from PromoCell (Heidelberg, Germany). The HUVEC were cultured in EGM, which was supplemented with supplements and 1% antibiotic–antimycotic. For all *in vitro* experiments, the cells were cultured in an incubator (Vision Bionex, Gyeonggi, Korea) containing 5% CO_2_ at 37 °C.

### 2.3 hASC-EVs isolation

hASC-EVs were isolated and characterized by the MISEV guideline, as provided by the International Society for Extracellular Vesicles [23,24]. When the hASC reached 80% confluence in the T-175 flask, the culture medium was removed, washed twice with PBS, and replaced with FBS-free conditioned medium. After 24 h of incubation, the conditioned medium of hASC was harvested, subjected to centrifugation (1500 rpm, 20 min, 4 °C), and filtered through a 0.22-μm membrane to remove cell fragments. The isolation of hASC-EVs was then undertaken using a capsule for tangential flow filtration (TFF, MWCO = 500 kDa; Pall Corporation, Port Washington, NY, USA), followed by dispersion in PBS.

### 2.4 Characterization of hASC-EVs

The size and distribution of hASC-EVs were characterized using nanoparticle tracking analysis (NTA, NanoSight LM10, Malvern, England). The NTA capture and analysis settings were as follows: capture duration, 30 s; shutter speed, 30 ms; viscosity, 1.0 c; camera level, 16; detection threshold, 5; screen gain, 10. The morphology of hASC-EVs was examined using a JEM-2100 F transmission electron microscope (TEM, JEOL, Tokyo, Japan) operating at 200 kV. To prepare the TEM samples, hASC-EVs were stabilized with a paraformaldehyde solution (2%) for 5 min, and the samples were deposited onto a carbon-film-coated grid with a 200-mesh parameter. For negative staining, hASC-EVs were subjected to a wash with DIW before incubation with a 1% solution of uranyl acetate for 1 min. To confirm the expression of EV protein markers, Western blot analysis was performed using the following primary antibodies: *anti*-Calnexin antibody (1:1000, ZRB1147, Sigma-Aldrich), anti-CD9 antibody (sc-13118, 1:1000, Santa Cruz Biotechnology, Dallas, TX, USA), *anti*-ALIX antibody (sc-53450, 1:200, Santa Cruz Biotechnology), *anti*-TSG101 antibody (sc-7964, 1:100, Santa Cruz Biotechnology), and *anti*-β-actin antibody (A5441, 1:5000, Sigma-Aldrich). The separation of proteins was achieved through the 10% sodium dodecyl sulfate-polyacrylamide gel electrophoresis (SDS-PAGE) procedure. To detect the primary antibodies, horseradish peroxidase-conjugated anti-mouse (31,430, 1:5000, Invitrogen, Waltham, MA, USA) or anti-rabbit IgG antibody (1460, 1:5000, Invitrogen) was used, and the signals were observed by a LAS-3000 device (Fujifilm, Tokyo, Japan). The molecular weights of proteins were distinguished using Precision Plus Protein Dual Colour Standards (Bio-Rad, Hercules, CA, USA).

### 2.5 Preparation of EGEV-Gel

Sodium alginate and CaCl_2_ were separately dissolved in DIW to prepare a 2% (w/v) alginate solution and a 2% (w/v) CaCl_2_ solution, respectively. Alginate and CaCl_2_ solutions were added into the mold at a 4:1 ratio, mixed thoroughly, and incubated at 37 °C for 1 h. Thereafter, the newly formed hydrogel was immersed in CaCl_2_ solution for 30 min before washing three times in DIW to remove excessive CaCl_2_. The hydrogel was dried in a clean bench at room temperature (RT). The hydrogel patches, composed of alginate alone (Gel), those containing either hASC-EVs (EV-Gel) or EGCG (EG-Gel), and those containing both components (EGEV-Gel), were prepared by rehydrating the dried hydrogels with 100 μL of PBS 1× (pH 7.4) only, 100 μL of PBS containing hASC-EVs (10^9^ particles), 100 μL of PBS containing EGCG (15 nmol), or a combination of both, respectively. The hydrogels were dried again in a clean bench at RT. The final hydrogel patch had a cylindrical shape (10 mm in diameter, 1 mm in thickness), and this patch was used in all subsequent experiments.

The morphology of the hydrogel patch was analyzed using a scanning electron microscope (SEM) by a model JSM-7600 F microscope (Hitachi, Tokyo, Japan) operating at 30 kV. For the sample preparation, the hydrogel patch was bisected, fully rehydrated, and subsequently freeze-dried. To evaluate the distribution of hASC-EVs within the EGEV-Gel, alginate was labeled with fluorescein, and hASC-EVs were stained with Flamma 675 NHS ester (BioActs, Incheon, Korea).

### 2.6 Characterization of hydrogel patch

Dried samples with an initial weight (W_0_) were immersed in 2 mL of PBS at 37 °C. At predetermined time points, the rehydrated hydrogels were removed from the PBS and weighed (W_t_). The swelling ratio of the sample was calculated as follows:

Swelling ratio = (W_t_ - W_0_) / W_0_ × 100%

Rehydrated hydrogel samples with an initial weight (W_0_) were immersed in 2 mL of PBS at 37 °C. At predetermined time points, the hydrogels were removed from the PBS and weighed (W_t_). The decomposition ratio of the sample was calculated as follows:

Decomposition ratio = (W_0_ - W_t_) / W_0_ × 100%

The *in vivo* degradation profile of the hydrogels was evaluated using a mouse model. At 0.5, 1, 3, 12, 24, 48, and 72 h after treatment, both the hydrogel patch and Tegaderm were removed, and the remaining hydrogel was collected and weighed.

To characterize EVs released from the hydrogel, hASC-EVs and EGEV-Gel were incubated in 2 mL of PBS at 4 °C for 2 days. To characterize the EVs released thereafter, their particle size, concentration, and size distribution were analyzed using NTA.

The rheological properties of hydrogel patches (10 mm in diameter, 1 mm in thickness) were examined using an ARES-G2 Rheometer (DE, USA). The rheometer was equipped with a parallel plate (25 mm in diameter), and the sample gap size was 0.35 mm. The storage modulus (G′) and loss modulus (G″) were determined while applying 1% shear strain at RT. The collected data were analyzed using the RheoCompass software. For the mechanical property, cylindrical patch samples (10 mm in diameter, 1 mm in height; *n* = 3, each group) were prepared and tested using a Universal Testing machine (Instron® 5566, Instron Corporation, Massachusetts, USA), where they were compressed to 60% of the whole thickness under 1.0 mm/min speed. The stress-strain curves were obtained, and the compressive strength was calculated using Bluehill® 2 software.

### 2.7 Antioxidant effect

The ability to scavenge free radicals was evaluated using the DPPH assay. The hydrogel patch was immersed and incubated in 2 mL of PBS for 48 h. Next, 2 mL of ethanol solution containing DPPH (200 μM) was mixed with the resulting PBS and incubated at 37 °C in the dark for 30 min. The absorbance of the control (DPPH solution without sample, $A_{\text{control}}$) and the sample ($A_{\text{sample}}$) was measured at 517 nm using a spectrophotometer (Agilent 8453 UV–Visible Spectroscopy System, Agilent Technology, USA). The scavenging rate (%) was calculated as follows:

Scavenging rate = (A_Control_ – A_Sample_) / A_Control_ × 100%

To quantify intracellular ROS levels *in vitro*, normal HDFs (nHDF) were seeded in a 6-well plate (2 × 10^5^ cells/well) for 24 h. The medium was then replaced with 2 mL of DMEM containing 1% FBS and 200 μM H_2_O_2_ for 12 h to induce damaged HDFs (dHDF). Next, the hydrogel patch was placed on 0.4-μm Polycarbonate (PC) Membrane Inserts and incubated with the cells for 2 days. To identify ROS, the cells were stained with DCF-DA (20 μM) for 20 min. After washing twice with PBS, the cells were incubated in PBS containing 0.01% w/v NaN_3_ and 2% v/v FBS (conditioned buffer). ROS scavenging was analyzed using a flow cytometer (Guava easyCyte system, EMD Millipore, Germany). The resulting data were also quantified using the FlowJo™ software (BD Life Science, Franklin Lakes, NJ, USA).

### 2.8 Evaluation of *in vitro* M1 inhibition

To evaluate the M1-inhibitory effect of EGEV-Gel, RAW 264.7 cells (considered as M0) were seeded at a density of 2 × 10^5^ cells/well in a 6-well plate and incubated in RPMI (10% FBS) at 37 °C for 24 h. Medium was replaced with RPMI (1 % FBS, IFN-γ (20 ng/mL), LPS (500 ng/mL)) to induce M1 macrophage polarization. A hydrogel patch was placed on 0.4-μm PC Membrane Inserts and incubated with the cells for 2 days. The cells were then washed twice with a conditioned buffer consisting of PBS containing 0.01% (w/v) NaN_3_ and 2% (v/v) FBS, followed by incubation with TruStain FcX (BioLegend Inc., San Diego, CA, USA) for 10 min. Cells were subsequently stained with PerCP anti-mouse CD86 antibody (105,025, 1:80, BioLegend Inc.) at 4 °C for 30 min. Isotype controls were stained with PerCP-rat IgG2a κ isotype antibody (400,529, 1:80, BioLegend Inc.). Finally, cells were washed twice with conditioned buffer and analyzed using a Guava easyCyte flow cytometry system. To confirm the expression of Nrf2 protein on RAW 264.7 cells, Western blot analysis was performed using the following primary antibodies: anti-ꞵ-actin antibody (A5,441, 1:5,000, Sigma-Aldrich) anti-Nrf2 antibody (1:1,000, PA5-27,882, Invitrogen). The separation of proteins was achieved through the 10% SDS-PAGE procedure. To detect the primary antibodies, horseradish peroxidase-conjugated anti-mouse (31,430, 1:5,000, Invitrogen) or anti-rabbit IgG antibody (1,460, 1:5,000, Invitrogen) was used, and the signals were observed by a LAS-3000. The molecular weights of proteins were distinguished using Precision Plus Protein Dual Colour Standards.

To evaluate the M1-inhibitory effect in primary cells, Bone marrow-derived macrophages (BMDMs) were induced from bone marrow cells were harvested from the leg bones of 7-week-old BALB/c mice. The isolated bone marrow cells were seeded with recombinant murine M-CSF (20 ng/mL^−1^) in RPMI (10% FBS) at 37 °C for 7days. To induce M1 polarization, BMDMs were grown with IFN-γ (20 ng/mL), LPS (500 ng/mL). The cells were stained with FITC anti-mouse F4/80 Recombinant Antibody (157,310, 1:80, BioLegend Inc.) and PE/Dazzle™ 594 anti-mouse CD86 Antibody (105,041, 1:80, BioLegend Inc.) at 4 °C for 30 min. Isotype controls were stained with FITC anti-rat IgG1 Antibody (406,001, 1:80, BioLegend Inc.) and PE/Dazzle™ 594 Rat IgG2a, κ Isotype Ctrl Antibody (400,557, 1:80, BioLegend Inc.). Cytokine expression in the culture supernatants of BMDMs was analyzed by enzyme-linked immunosorbent assay (ELISA). Collected media were centrifuged at 300 × g for 5 min at 4 °C to obtain the supernatants. The levels of TNF-α and IL-10 were then quantified using ELISA kits according to the manufacturer's instructions.

### 2.9 Migration property

To confirm the wound healing ability of EGEV-Gel, a scratch assay was performed. In brief, nHDFs were seeded in a 6-well plate at a density of 2 × 10^5^ cells/well and cultured until they reached approximately 95% confluence. Subsequently, scratches were created using a 200 μL pipette tip, and cells were washed with PBS. The medium was replaced with DMEM containing 1% FBS and 200 μM H_2_O_2_ to generate dHDFs. Afterward, the hydrogel patch was placed on 0.4-μm PC Membrane Inserts and incubated with the cells for 2 days. During incubation, cell migration was monitored using a camera connected to a microscope, and the images were analyzed using the ImageJ software.

### 2.10 Cytotoxicity

nHDFs, dispersed in DMEM containing 10% FBS, were seeded separately in a 6-well plate (2 × 10^5^ cells/well), followed by incubation in a CO_2_ incubator for 24 h. The medium was then replaced with DMEM containing 1% FBS with 200 μM H_2_O_2_, and the cells were incubated for 12 h to generate dHDF. Thereafter, the hydrogel patch was incubated with the cells for 2 days using 0.4-μm Pore PC Membrane Inserts. At the end of the incubation, 3-(4,5-dimethylthiazol-2-yl)-2,5-diphenyltetrazolium bromide (MTT) reagent (500 μg/mL) was added to each well and incubated at 37 °C for 2 h. After the removal of the MTT-containing medium, 100 μL of dimethyl sulfoxide was added to each well to dissolve the violet formazan crystals. The optical density of each well was measured using a microplate reader at a wavelength of 570 nm.

### 2.11 In vitro tube formation assay

Normal HUVEC cells (nHUVEC), dispersed in EGM, were separately seeded in a 6-well plate (2 × 10^5^ cells/well), followed by incubation in a CO_2_ incubator for 24 h. For the capillary-like tube formation assay, growth factor-depleted Matrigel (BD Pharmingen, CA, USA) was applied at 500 μL to each well. Following the polymerization of the Matrigel at 37 °C for 24 h, cells were treated with H_2_O_2_ to induce damaged HUVEC (dHUVEC) and then treated with samples. Finally, images of the cell vascularization were observed using a camera connected to a microscope.

### 2.12 Animal experiments

For the *in vivo* experiments, eight-week-old female Balb/c mice were purchased from Orient Bio Inc. (Seoul, Republic of Korea). All animal experiments in this research were carried out in strict accordance with relevant ethical regulations and protocols approved by the Institutional Animal Care and Use Committee (IACUC) of Sungkyunkwan University (SKKUIACUC2024-04-34-1) and were conducted following institutional guidelines. The wound model was established using eight-week-old female Balb/c mice (20–25 g body weight). Mice were anesthetized using a mixture of Zoletil and Rompun, after which two full-thickness circular wounds (10 mm in diameter) were surgically created on the dorsal skin of each mouse. Then, mice were randomly subdivided into five groups (*n* = 8 per group): Tegaderm, Gel, EG-Gel, EV-Gel, and EGEV-Gel. Hydrogel patch was applied to the wound sites and covered with a commercial dressing (Tegaderm; 3M Healthcare, St. Paul, MN, USA). After 3 days of treatment, both the hydrogel patch and Tegaderm were removed, and the wound healing process and body weight were monitored over 12 days. Wound images were captured every 3 days using a digital camera and analyzed using ImageJ software. To confirm *in vivo* ROS scavenging and M1 macrophage population, skin tissues were harvested on day 3. For the evaluation of skin regeneration efficacy, additional tissue samples were collected on day 12. The harvested skin tissues were then fixed and stored for further analysis.

### 2.13 Histological and immunofluorescent analysis

Formalin-fixed, paraffin-embedded tissue samples were sectioned at a thickness of 4 μm and stained with either H&E or the Masson's trichrome (MT) staining kit. Stained sections were imaged using the Axio Scan Z1 Slide scanner (Carl Zeiss, Oberkochen, Baden-Wurttemberg, Germany). For immunohistochemistry (IHC), paraffin-embedded skin tissue sections were deparaffinized, blocked, and incubated with the respective primary antibodies. Subsequently, the nuclei were stained with 4′,6-diamidino-2-phenylindole solution, and superoxide levels were visualized using dihydroethidium (DHE, Invitrogen). Stained sections were then observed using a CLSM. The following antibodies were utilized in this study: F4/80 Monoclonal Antibody (14-4801-81, 1:50, Invitrogen), Mouse anti-Rat IgG2a Secondary Antibody eFluor™ 615 (42-4817-80, 1:100, Invitrogen), CD86 Polyclonal Antibody (PA5-114,995, 1:100, Invitrogen), and Goat anti-Rabbit IgG (H + L) Cross-Adsorbed Secondary Antibody Alexa Fluor™ 488 (A-11008, 1:100, Invitrogen) were used to visualize M1 macrophage; Fibroblasts Monoclonal Antibody (ER-TR7) (MA1-40076, 1:50, Invitrogen) was used to visualize fibroblasts; Cytokeratin 19 Antibody (A-3) Alexa Fluor® 647 (sc-376,126 AF647, 1:100, Santa Cruz Biotechnology) was used to visualize hair follicle; CD31 (PECAM-1) Monoclonal Antibody (390), FITC (14-0311-81, 1:100, Invitrogen), and Alpha-Smooth Muscle Actin Monoclonal Antibody (1A4), Alexa Fluor™ 488 (53-9760-82, 3 μg/mL, Invitrogen) were used to visualize angiogenesis; anti-Nrf2 antibody (1:1,000, PA5-27,882, Invitrogen) together with Goat anti-Rabbit IgG (H+L) Cross-Adsorbed Secondary Antibody Alexa Fluor™ 488 (A-11,008, 1:100, Invitrogen) were used to visualize Nrf2 expression.

### 2.14 Statics

Data was presented as mean ± SD for all experiments. For comparisons between two groups, an unpaired two-tailed Student's t-test was used. For comparisons involving a single factor, one-way analysis of variance (ANOVA) followed by Tukey's post hoc test was applied. For experiments involving two independent variables, two-way ANOVA followed by appropriate multiple comparisons testing was performed. A p-value <0.05 was considered statistically significant.

## 3 Results and discussion

### 3.1 Preparation and characteristics of EGEV-Gel

**Fig. 2:**
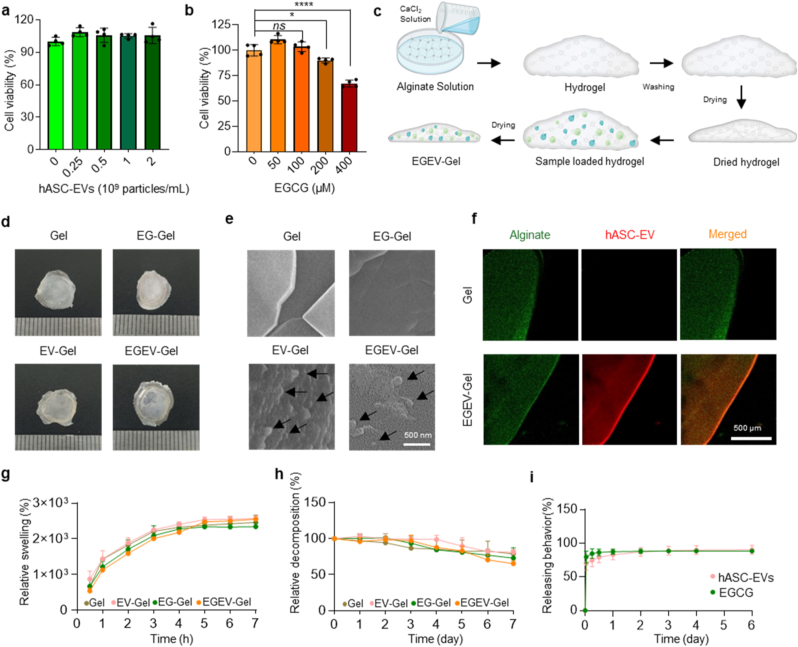
**Characteristics of EGEV-Gel.** (a) Cytotoxicity of hASC-EVs (n = 4). (b) Cytotoxicity of EGCG (n = 4). (c) Schematic illustration depicting the preparation of EGEV-Gel. (d) Representative images of dried hydrogel patches. (e) SEM image of dried hydrogel patches. (f) hASC-EVs distribution in EGEV-Gel patch. (g) Swelling property of dried hydrogel patches (n = 3). (h) Decomposition property of dried hydrogel patches (n = 3). (i) Release behavior of hASC-EVs and EGCG from rehydrated EGEV-Gel patch. Error bars represent the ±SD. Statistical significances were calculated using the one-way ANOVA method (∗p < 0.05, ∗∗p < 0.01, ∗∗∗p < 0.001, and ∗∗∗∗p < 0.0001).

Before being incorporated into the hydrogel, the characteristics of hASC-EVs and EGCG were confirmed. The hASC-EVs exhibited a spherical morphology with an average size of 130 nm (Fig. S1a and b). Moreover, they expressed typical biomarkers of small EVs such as CD9 and TSG101 (Fig. S1c), indicating successful isolation in accordance with the MISEV guidelines [23,24]. Moreover, the particle-to-protein ratio of hASC-EVs remained consistent across batches (Fig. S1d), consistent with previous reports [25]. The cytotoxicity of hASC-EVs was also evaluated using HDFs, and no cytotoxic effects were observed across the range of concentrations tested (Fig. 2a). In the case of EGCG, which exhibited a strong absorption at 275 nm (Fig. S2) due to its polyphenolic structure [26,27], it was also found to be non-cytotoxic to HDFs at concentrations up to 100 μM (Fig. 2b), consistent with previous findings [28]. Based on preliminary dose–response studies, the concentrations were selected within biologically effective ranges for experiments (Fig. S3).

The hydrogel patches, prepared as illustrated in Fig. 2c, exhibited a translucent appearance, and their morphology was not significantly altered by the incorporation of EGCG or hASC-EVs (Fig. 2d). The hydrogels demonstrated stable adhesion to human skin, maintaining effective contact throughout the treatment period maintaining effective contact throughout the treatment period (Fig. S4). Cross-sectional SEM images of the patches (Fig. 2e–S5) revealed numerous nanovesicle-like structures (50–150 nm) on the surfaces of EV-Gel and EGEV-Gel, as indicated by black arrows. Moreover, the fluorescent images in Fig. 2f indicated that hASC-EVs were evenly distributed inside the hydrogel patch. All the hydrogel patches could absorb a quantity of water, thus reaching a swelling ratio of approximately 1000% within 1 h (Fig. 2g). This property offers a significant advantage, as it enables the hydrogel patch to rapidly absorb wound exudate, helping to maintain a clean and moist wound environment - an essential factor for promoting efficient healing and reducing the risk of infection in clinical settings.

The degradation of the hydrogel patches was also assessed by monitoring their weight in PBS over time (Fig. 2h). No significant differences were observed among the groups, indicating that the incorporation of hASC-EVs or EGCG did not noticeably affect the degradation behavior of the hydrogel patch [29]. In addition, *in vivo* degradation analysis showed that the hydrogel degraded substantially within 72 h (Fig. S6), likely due to enzymatic activity and the dynamic wound microenvironment. This behavior was consistent with the release profile of hASC-EVs and EGCG over 144 h (Fig. 2i–Table S1), with most of the payload delivered within the first 48 h, ensuring effective therapeutic delivery. This profile aligned with the early inflammatory phase of wound healing, during which ROS accumulation and pro-inflammatory macrophage activity are most prominent. Batch-to-batch confirmed using independent EGEV-Gel preparations, which exhibited comparable particle release by NTA (Fig. S7). The integrity of EVs after release was preserved, with no significant changes in the characteristics of hASC-EVs (Fig. S8). Collectively, these results demonstrate reproducible delivery while maintaining structural integrity. Finally, the rheology results show that the storage modulus (G′) remained stable and consistently higher than the loss modulus (G″), confirming the elastic-dominant behavior of EGEV-Gel Gel (Fig. S9). Also, the alginate network preserved its structural integrity without significant weakening or erosion. This indicates that the release of EVs and EGCG from EGEV-Gel is governed primarily by diffusion rather than erosion.

### 3.2 Anti-inflammatory and regenerative function of EGEV-Gel

**Fig. 3:**
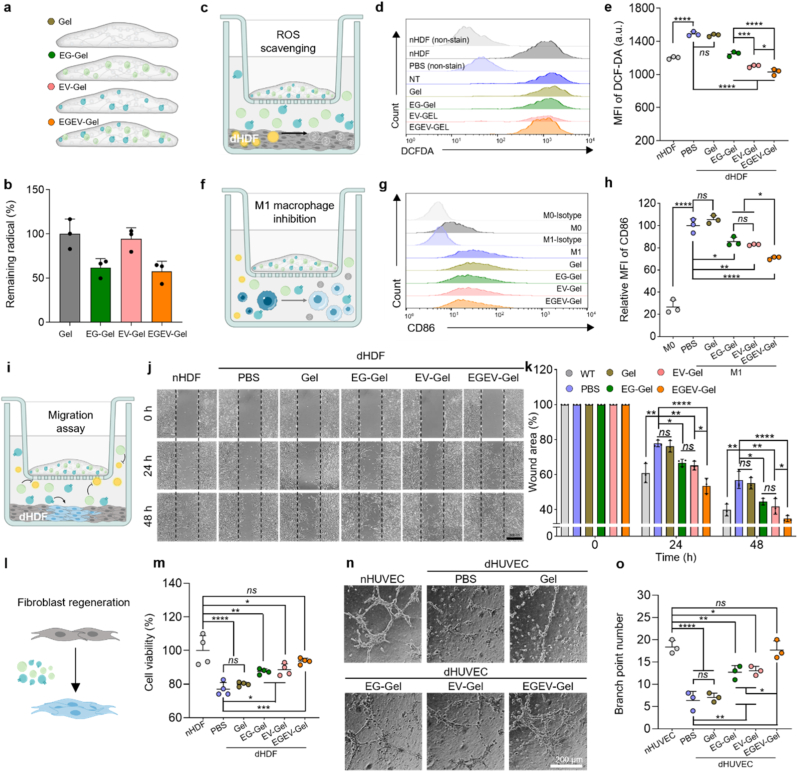
**In vitro effects of EGEV-Gel in ROS scavenging and M1 inhibition.** (a) Schematic illustration depicting the structure of each hydrogel patch. (b) Free radical degradation of hydrogel patches (n = 3). (c) Schematic illustration of ROS scavenging. (d) Representative flow cytometry plots showing ROS in dHDFs with different treatments and (e) related quantification (n = 3). (f) Schematic illustration of M1 macrophage inhibition. (g) Representative flow cytometry plots showing CD86 expression in M1 macrophage after different treatments and (h) related quantified result (n = 3). (i) Schematic illustration of the Migration assay. (j) Representative images of dHDFs migration (scale bar = 500 μm) and (k) quantitative result (n = 3). (l) Schematic illustration of Fibroblast regeneration. (m) Viability of dHDFs after different treatments (n = 4). (n) Representative image of tube formation by HUVEC cells (scale bar = 200 μm) with (o) related branch point number (n = 3). Error bars represent the ±SD. Statistical significances were calculated using the one-way ANOVA method (∗p < 0.05, ∗∗p < 0.01, ∗∗∗p < 0.001, and ∗∗∗∗p < 0.0001).

EGCG, a polyphenol, reduces ROS by directly neutralizing free radicals and upregulating endogenous antioxidant enzymes [30], whereas the antioxidant effects of hASC-EVs mainly depend on the activation of endogenous antioxidant enzymes [[31], [32], [33]]. Based on these complementary mechanisms, the combination of hASC-EVs and EGCG was expected to exert synergistic effects in scavenging ROS. To assess radical scavenging activity, the hydrogel patches, prepared under different conditions (Fig. 3a), were first evaluated using the DPPH assay. As expected, EGCG-containing EG-Gel effectively reduced free radical levels, whereas EV-Gel containing only hASC-EVs showed no significant scavenging activity (Fig. 3b–S10). We next examined the intracellular ROS scavenging effects of the hydrogel patches by exposing them to dHDFs (Fig. 3c–e). Both EG-Gel and EV-Gel significantly suppressed intracellular ROS levels, implying their intrinsic antioxidant properties. Notably, EGEV-Gel exhibited a markedly greater reduction in intracellular ROS levels compared to either EG-Gel or EV-Gel alone, confirming a synergistic antioxidant effect of hASC-EVs and EGCG.

To evaluate the function of EGEV-Gel on inhibiting inflammatory immune cells, hydrogel patches were incubated with RAW264.7 cells, pretreated with IFN-γ^+^ and LPS^+^ to induce M1 macrophages [7]. The result indicated that compared with the PBS group, the EG-Gel and EV-Gel groups slightly reduced the expression of CD86 (M1 biomarker) (Fig. 3f–h, S11). Notably, owing to the synergistic effect of EGCG and hASC-EVs, the EGEV-Gel group dramatically down-regulated the CD86 expression compared to either EG-Gel or EV-Gel alone. In contrast, the Gel group exhibited no effect on CD86 expression, indicating that the observed M1 macrophage inhibition was specifically attributable to hASC-EVs and EGCG. These findings were further confirmed in BMDM, where EGEV-Gel significantly reduced CD86 expression (Fig. S12), demonstrating a synergistic effect of hASC-EVs and EGCG in suppressing M1 macrophage polarization. Additionally, the anti-inflammatory effects were validated at the molecular level by evaluating the concentrations of TNF-α and IL-10 released from BMDMs (Fig. S13). EGEV-Gel downregulated pro-inflammatory signaling (TNF-α) while enhancing anti-inflammatory responses (IL-10).

The recovery of cellular functions was further evaluated by assessing HDF migration and viability, as well as endothelial tube formation by HUVEC. In scratch assays, the migration of dHDF, cultured with EGEV-Gel, was remarkably enhanced compared with all other groups (Fig. 3i–k), indicating substantial restoration of fibroblast function. When damaged by H_2_O_2_, the viability of HDFs was dramatically reduced to 77.2%, compared with that of nHDFs (Fig. 3l and m). Treatment with EG-Gel and EV-Gel significantly improved cell viability to 88.2% and 90.0%, respectively, while EGEV-Gel achieved the highest recovery at 94.1%. Notably, the viability in the EGEV-Gel group was comparable to that of the nHDF group, suggesting the excellent property of EGEV-Gel for restoring the function of dHDF. Endothelial function was also examined using tube formation assays with dHUVECs. Co-culture with EGEV-Gel resulted in a greater number of branch points than PBS, EG-Gel, or EV-Gel treatments (Fig. 3n and o). Strikingly, tube formation in the EGEV-Gel group approached that of nHUVECs, implying a strong capability of EGEV-Gel in recovering endothelial cell function.

### 3.3 Wound healing effects of EGEV-Gel *in vivo*

**Fig. 4:**
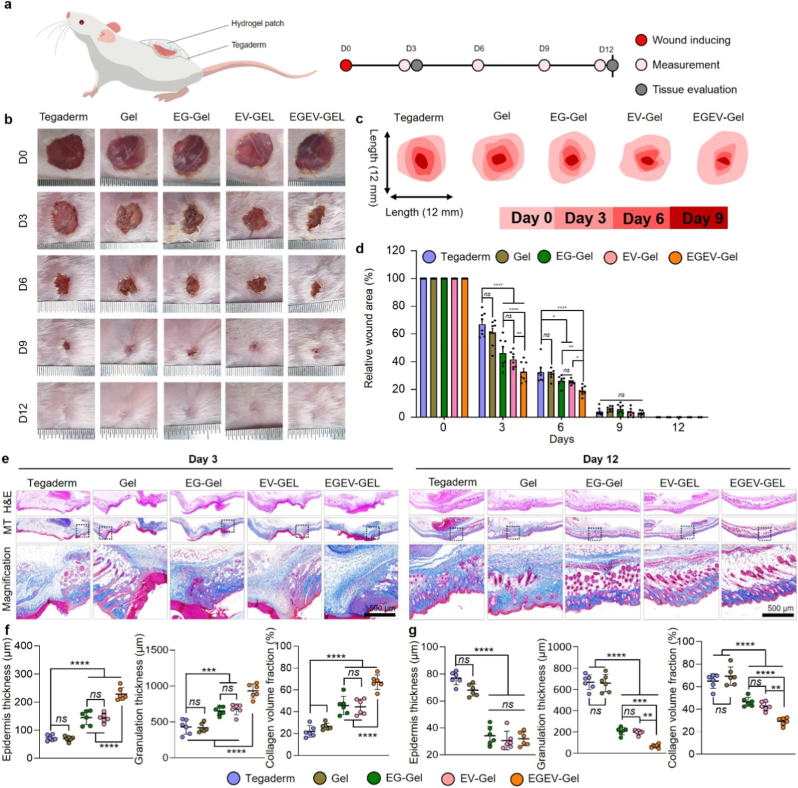
**Therapeutic effects of EGEV-Gel in a wound healing mouse model.** (a) Schematic illustration of the treatment regimen of each hydrogel patch. (b) Representative images of the wound area at different time points. (c) Traces of wound closure. (d) Quantitative analysis of relative wound area over the period of treatment (n = 8). (e) H&E staining and Masson's trichrome staining for the wound area after days 3 and 12. Quantification of (f) epidermis thickness, granulation thickness, and collagen volume fraction in the wound on day 3 (n = 6). (g) Quantification of epidermis thickness, Granulation thickness, and collagen volume fraction after wound healing (day 12) (n = 6). Error bars represent the ±SD. Statistical significance values were calculated via one-way ANOVA for (e), (f), and (g), while one-way ANOVA for (d) (∗p < 0.05, ∗∗p < 0.01, ∗∗∗p < 0.001, and ∗∗∗∗p < 0.0001).

Given the anti-inflammatory and regenerative properties of EGEV-Gel, it was expected to be beneficial for wound treatment. To evaluate its wound-healing potential, circular wounds were surgically created on the dorsal skin of mice, and wound areas were measured every three days over a 12-day period (Fig. 4a). Compared with the control group treated with Tegaderm alone, the Gel group showed no significant effect on wound repair (Fig. 4b–d). In contrast, wound healing was noticeably enhanced in the EG-Gel and EV-Gel groups, with the most pronounced improvement observed in the EGEV-Gel group. In particular, at both day 3 and day 6, the EGEV-Gel consistently showed the highest function for repairing the wound, supporting a synergistic contribution of hASC-EVs and EGCG to the wound-healing process. These *in vivo* results are consistent with the earlier *in vitro* findings (Fig. 3), where EGEV-Gel demonstrated superior ROS scavenging capacity and more effectively restored fibroblast and endothelial cell functions, compared to either EG-Gel or EV-Gel alone. The combined antioxidant activity of EGCG and the regenerative, anti-inflammatory properties of hASC-EVs likely acted in concert to reduce oxidative stress, suppress excessive inflammation, and accelerate tissue regeneration, ultimately leading to more rapid and complete wound closure.

To further assess the effects of patch treatment on skin architecture during wound healing, tissue samples were collected on days 3 and 12 for histological analysis using H&E staining, MT staining, and IHC. On day 3, the epidermis thicknesses, granulation tissue thicknesses, and collagen volume fraction in the EG-Gel and EV-Gel groups were significantly higher than those in the Tegaderm group (Fig. 4e and f). Notably, the EGEV-Gel group exhibited the highest values among all treatments, indicating that EGEV-Gel accelerated the healing process. Interestingly, the results observed on day 12 showed the opposite trend. During the remodeling phase, the epidermal thickness, granulation tissue thickness, and collagen volume fraction were markedly reduced in the EG-Gel and EV-Gel groups, compared with the Gel group. The most pronounced reduction was observed in the EGEV-Gel group (Fig. 4e–g). Furthermore, by day 12, both the morphology and structure of the regenerated skin in the EGEV-Gel group were comparable to those of the surrounding normal skin, implying the comprehensive wound-healing function of EGEV-Gel. In addition, no significant changes in body weight (Fig. S14) and negligible hemolytic activity (Fig. S15) further support the good *in vivo* and blood biocompatibility of the material. Collectively, these results elucidate the superior efficacy of EGEV-Gel in promoting wound repair, supporting its potential as a promising candidate for the treatment of acute wounds, such as those resulting from trauma or severe injury.

### 3.4 Restoration of skin architecture by EGEV-Gel *in vivo*

**Fig. 5:**
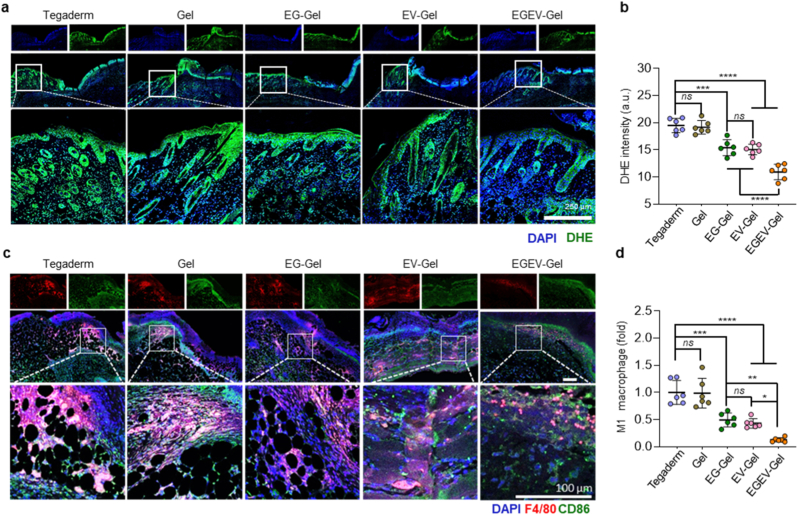
***In vivo* ROS scavenging and M1 inhibition of EGEV-Gel.** (a) The DHE staining on day 3 in wounded skin, and (b) Quantitative results of DHE (n = 6). (c) Macrophage staining on day 3 in wounded skin. (d) Quantitative results of CD86 with F4/80 (n = 6). Error bars represent the ±SD. Statistical significance values were calculated via one-way ANOVA (∗p < 0.05, ∗∗p < 0.01, ∗∗∗p < 0.001, and ∗∗∗∗p < 0.0001).

Excessive ROS accumulation and elevated M1 macrophage populations are known to delay or impair the wound healing process [[34], [35], [36]]. We hypothesized that EGEV-Gel could simultaneously downregulate ROS levels and reduce M1 macrophage infiltration *in vivo*. As expected, strong DHE fluorescence signals were observed in the Tegaderm and Gel groups on day 3, implying an excessive ROS accumulation during the inflammatory stage of the healing process (Fig. 5a and b). However, ROS signals were reduced slightly in the EG-Gel and EV-Gel groups and significantly diminished in the EGEV-Gel group, demonstrating its potent ROS-scavenging capacity. Besides, the M1 population (F4/80^+^ CD86^+^ cells) was remarkably reduced in the EGEV-Gel group compared with the Tegaderm and Gel groups, whereas EG-Gel and EV-Gel each showed a substantial effect on M1 levels (Fig. 5c and d). Quantitative analysis (Fig. 5b–d) further confirmed that EGEV-Gel dramatically reduced both ROS levels and M1 macrophage numbers relative to the Tegaderm group. These findings suggest that EGEV-Gel effectively mitigates oxidative stress and excessive inflammation, thereby supporting a more favorable wound healing environment.

**Fig. 6:**
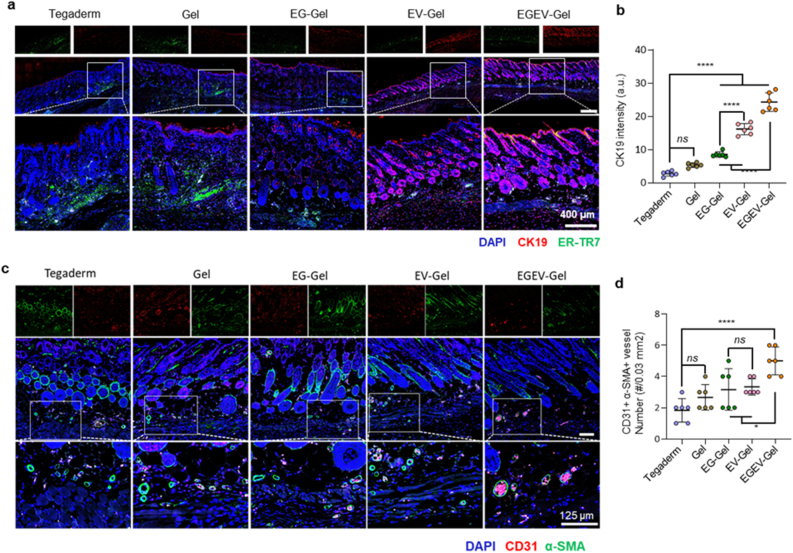
**EGEV-Gel supported hair follicle and vascular regenerations in the wound healing process.** (a) Fibroblasts were stained with ER-TR7 Ab, while hair follicles were stained with CK19 Ab. (b) Quantitative results of CK19 (n = 6). (c) Blood vessels were stained with CD31 Ab and α-SMA Ab. (d) Number of CD31+α-SMA + vessel counted from (c) (n = 6). Error bars represent ± SD. Statistical significance values were calculated via one-way ANOVA (∗p < 0.05, ∗∗p < 0.01, ∗∗∗p < 0.001, and ∗∗∗∗p < 0.0001).

We hypothesize that the synergistic effect of hASC-EVs and EGCG in the gel patch would exert a potent effect during the skin remodeling stage of wound healing. As shown in Fig. 6a and b, extensive and unlocalized fibroblasts (ER-TR7^+^ cells) were observed in the dermis of the Tegaderm and Gel groups, indicating an incomplete healing process. In contrast, fibroblast localization was markedly reduced in the EG-Gel, EV-Gel, and particularly the EGEV-Gel groups. Interestingly, in the EGEV-Gel group, fibroblasts were not only present in the dermis but also organized within the papillary layer, exhibiting a distribution pattern similar to that of normal skin. It should be noted that Cytokeratin 19 (CK19) is also commonly used as a marker for hair follicle stem cells [19] locating in the bulge region [37,38]. During the wound healing process, these CK19-positive HFSCs are known to exit the niche, migrate toward the wound bed, and contribute to re-epithelialization [39]. The data showed a slight increase in hair follicle signal in the EG-Gel group and a significant increase in the EV-Gel group, highlighting the notable role of hASC-EVs in promoting hair follicle regeneration (Fig. 6a and b). Remarkably, the EGEV-Gel group exhibited an even higher and more uniform signal than the EV-Gel group, indicating that EGEV-Gel could strongly restore skin function, including sensory perception and thermoregulatory properties [40]. It is worth noting that monitoring the other conditions of skin appendages around the wound, such as angiogenesis, can also serve as an indicator of the extent and advancement of wound healing [41]. The angiogenesis was noticed by CD31 and α-smooth muscle actin (α-SMA) staining [42], which is shown and quantified in Fig. 6c and d. The result indicated that the number of CD31^+^ α-SMA^+^ vessels in the dermis layer of the EGEV-Gel group was dramatically higher than in the other groups. To gain mechanistic insight, the Nrf2 signaling pathway was evaluated. EGEV-Gel treatment increased Nrf2 expression in both macrophages and wound tissues (Figs. S16 and S17), suggesting that Nrf2 activation contributes to the regulation of oxidative stress and the suppression of pro-inflammatory macrophage responses [43,44]. Given these regenerative effects, EGEV-Gel represents a promising strategy for enhancing wound healing.

## 4 Conclusion

In this study, we successfully developed EGEV-Gel, an alginate-based hydrogel patch containing hASC-EVs and EGCG, which covers the wound area and gradually releases bioactive components to exert synergistic effects. This system demonstrated remarkable *in vitro* efficacy in scavenging ROS, inhibiting M1 macrophages, and restoring the function of dHDFs. Furthermore, *in vivo* results revealed that EGEV-Gel accelerated wound closure, while supporting angiogenesis and hair follicle regeneration. Altogether, this innovative formulation provides a way for convenient and effective topical delivery of MSC-EVs and represents a promising therapeutic approach for wound healing.

## CRediT authorship contribution statement

**Won Ho Jang:** Formal analysis, Investigation, Methodology, Writing – original draft. **Van Dat Bui:** Formal analysis, Investigation, Methodology, Visualization, Writing – original draft. **Van Hieu Duong:** Investigation. **Sol Shin:** Investigation. **Jungmi Lee:** Investigation. **Torsha Ghosh:** Investigation. **Chang Hyun Lee:** Investigation. **Soyoung Son:** Investigation. **Jiyeon Kim:** Investigation. **Eun-Cheol Lee:** Investigation. **Suk Ho Bhang:** Investigation. **Jae Hyung Park:** Conceptualization, Funding acquisition, Resources, Supervision, Writing – review & editing.

## Declaration of Competing Interest

The authors declare that they have no known competing financial interests or personal relationships that could have appeared to influence the work reported in this paper.

## Data Availability

No data was used for the research described in the article.
