# Supplementary material for: Extracellular Vesicle-embedded alginate hydrogel patch for accelerated wound healing
Source: Mater Today Bio. 2026 May 27;38:103288. doi: 10.1016/j.mtbio.2026.103288 (PMC13264183; doi:10.1016/j.mtbio.2026.103288)
Supplement: Multimedia component 1 [file mmc1.docx]

**Supplementary data**


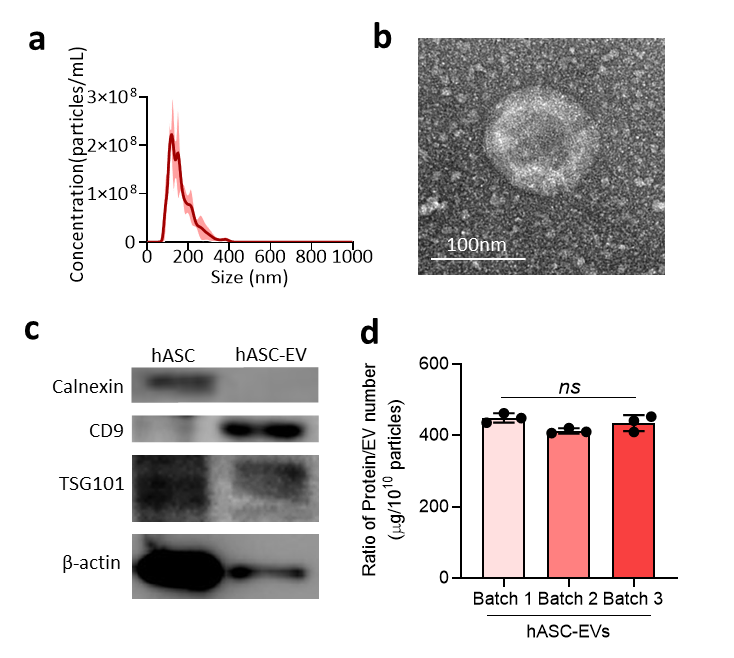


**Figure S1. Characteristic of hASC-EVs.** (a) Size distribution of hASC-EVs measured by NTA. (b) The TEM image of hASC-EVs. (c) Western blotting analysis for biomarkers of hASC-EVs and their parent cells. (d) Comparison of particle-to-protein ratio of hASC-EVs in 3 different batches (n = 3). Statistical significances were calculated using the one-way ANOVA method. Error bars represent the SD.


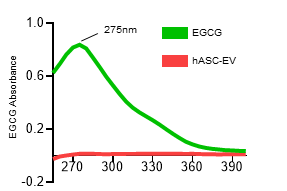


**Figure S2.** **Characteristic of EGCG Absorbance wavelength measured by UV-vis.**


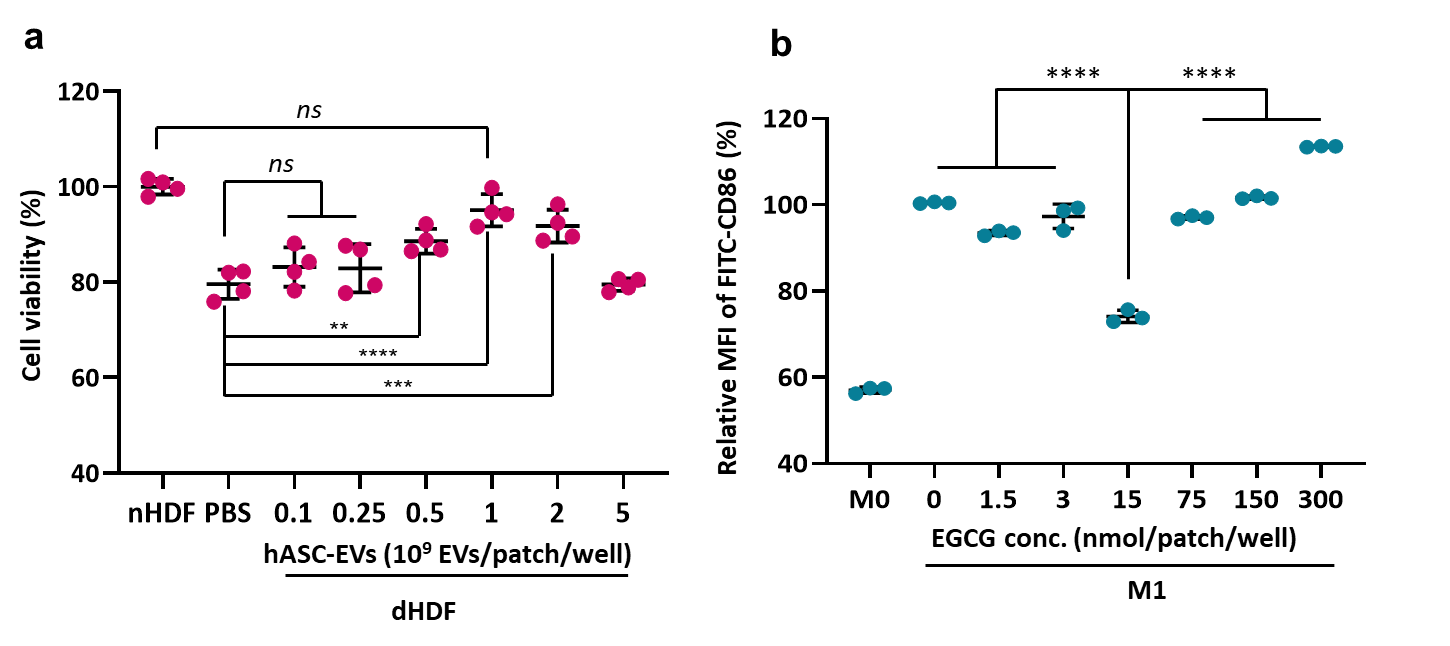


**Figure S3. Dose-dependent effects of hASC-EVs and EGCG.** (a) Cytotoxicity of hASC-EVs in dHDF at increasing concentrations (n=4). (b) Effect of EGCG on M1 macrophage polarization in RAW264.7 cells, assessed by CD86 expression (MFI) (n = 3). Statistical significances were calculated using the one-way ANOVA method (**p < 0.01, ***p < 0.001, and ****p < 0.0001). Error bars represent the SD.


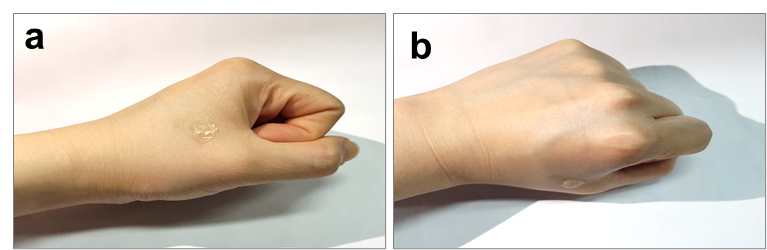


**Figure S4. Evaluation of skin adhesion properties of the dried EGEV-Gel patch on human skin.** (a) Application of the dried hydrogel patch onto moist human skin, followed by rapid hydration. (b) Formation of conformal adhesion after hydration, enabling stable attachment under hand movement.


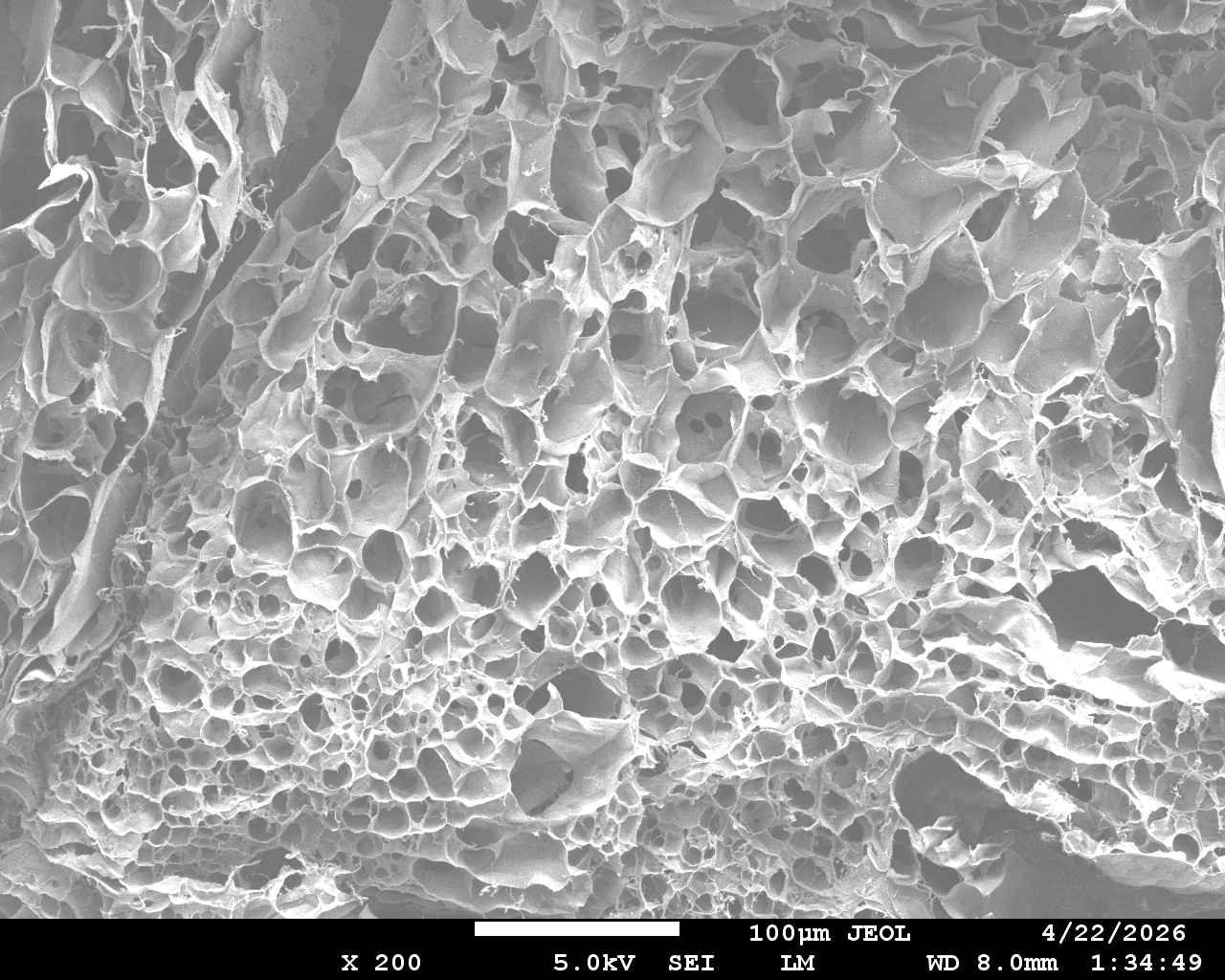


**Figure S5. Microstructural characterization of EGEV-Gel.** Representative SEM images showing the porous structure.

**Figure S6. In vivo degradation profile of hydrogels.** The remaining mass of Gel and EGEV-Gel was measured at different time points after application in the wound model (n =3). Error bars represent the SD.

**Figure S*7*. Batch-to-batch consistency of particle release from EGEV-Gel after 48 h.** The total number of hASC-EVs released from three independent batches of EGEV-Gel was quantified at 48 hours using NTA (n = 3). Statistical significances were calculated using the one-way ANOVA method. Error bars represent the SD.


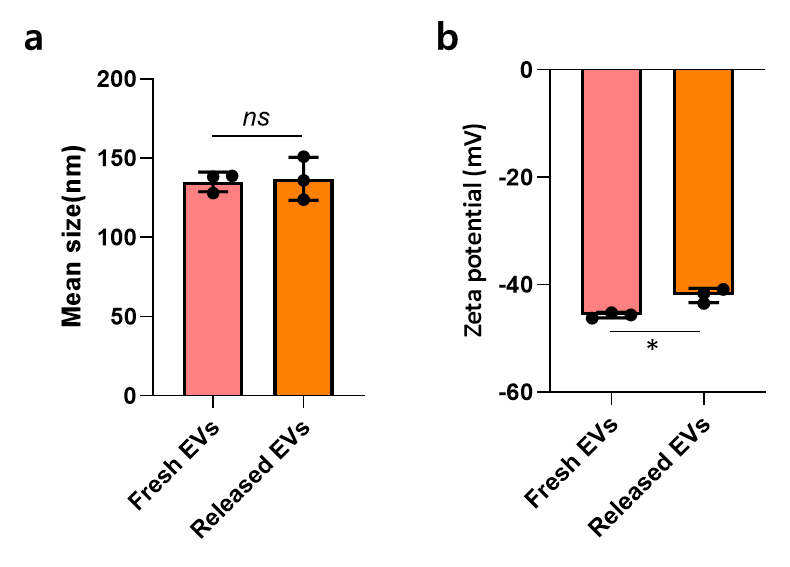


**Figure S8. Mean particle size of fresh and released EVs.** (a) The mean size and (b) zeta potential of hASC-EVs before loading (fresh EVs) and after released from EGEV-Gel (released EVs) was measured by NTA (n = 3). Statistical significances were calculated using unpaired two-tailed Student’s t-test. Error bars represent the SD.


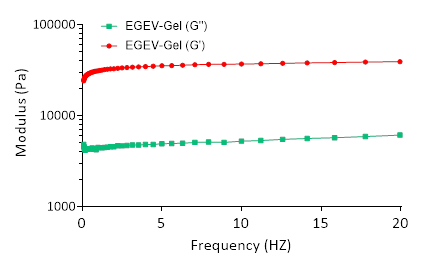


**Figure S9. Storage modulus (G′) and loss modulus (G″) of EGEV-Gel.**


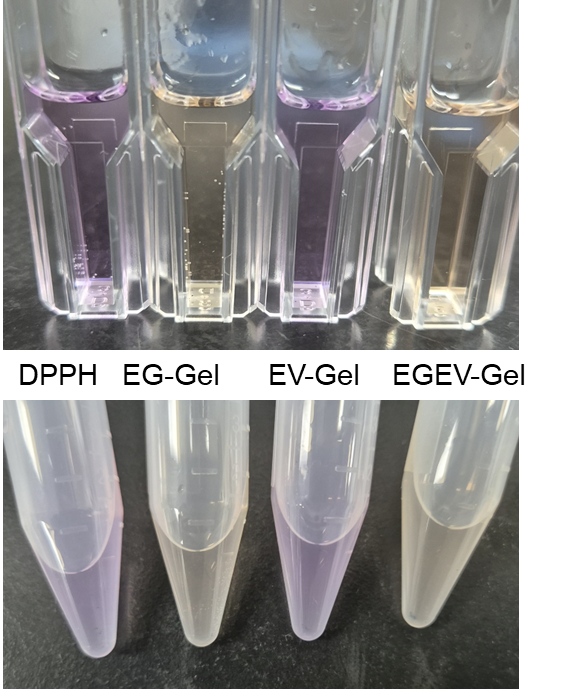


**Figure S10. Free radical degradation. Representative images of DPPH solution.**


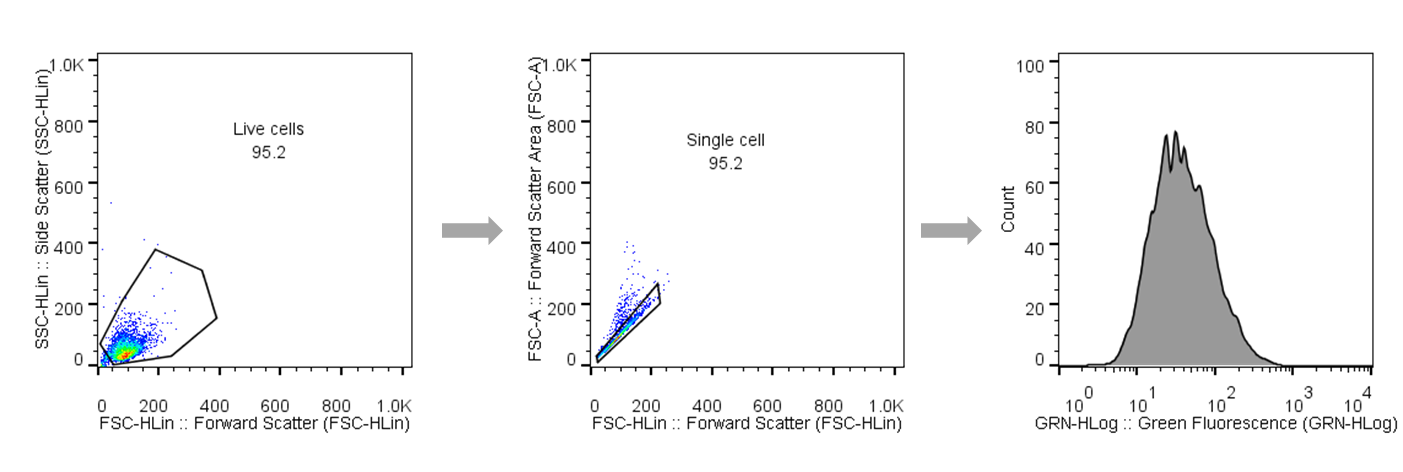


**Figure S11. Flow cytometry gating strategy for cell population analysis.** Representative gating workflow showing the selection of live cells, followed by singlet discrimination, and subsequent analysis of the target population based on fluorescence intensity. Percentages indicate the proportion of cells within each gated population.


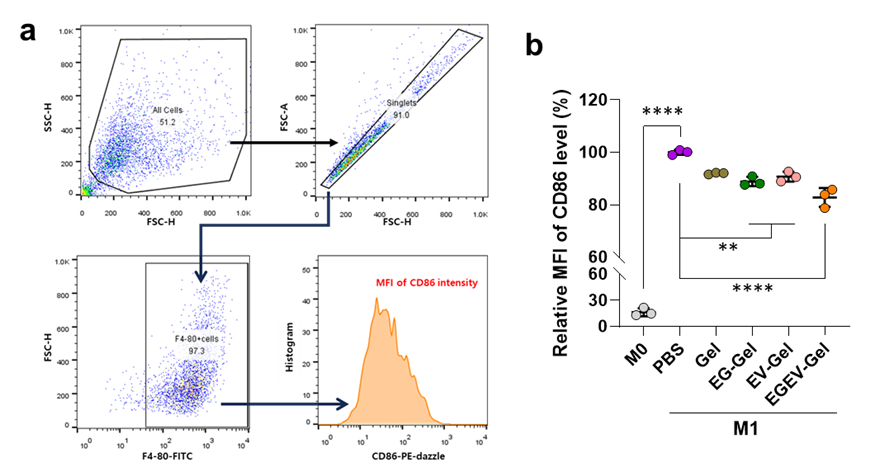


**Figure S12. Evaluation of M1 macrophage polarization in BMDMs.** (a)Gating strategy for flow cytometry analysis of M1 polarized BMDMs stimulated with LPS & IFN-γ. (b) Relative MFI of CD86 level expression in M1 macrophages after different treatments (n = 3). Statistical significances were calculated using the one-way ANOVA method (*p < 0.05, **p < 0.01, and ****p < 0.0001). Error bars represent the SD.


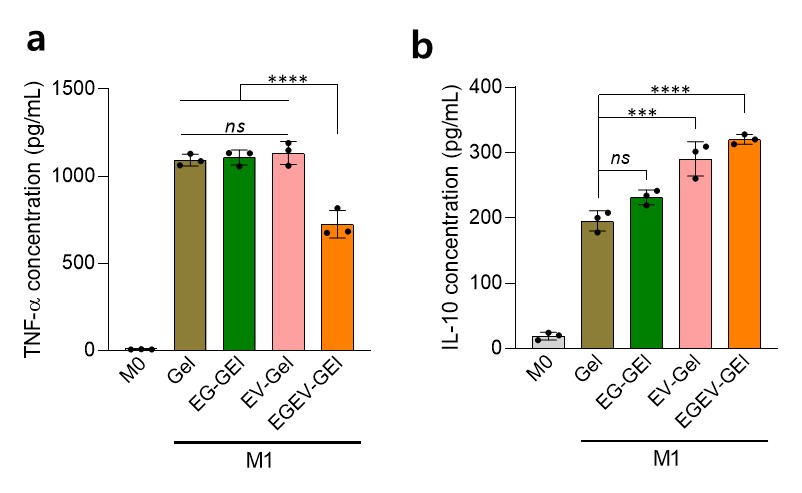


**Figure S13**. **Effects of EGEV-Gel in cytokine expression in BMDMs.** (a) TNF-α expression levels in LPS-stimulated BMDMs after different treatments were analyzed by ELISA. (b) IL-10 expression levels were analyzed by ELISA (n = 3). Statistical significances were calculated using the one-way ANOVA method (***p < 0.001, and ****p < 0.0001). Error bars represent the SD.

**Figure S14. Body weight changes during in vivo treatment.** Body weight of animals was monitored throughout the experimental period (n = 7). Error bars represent the SD.


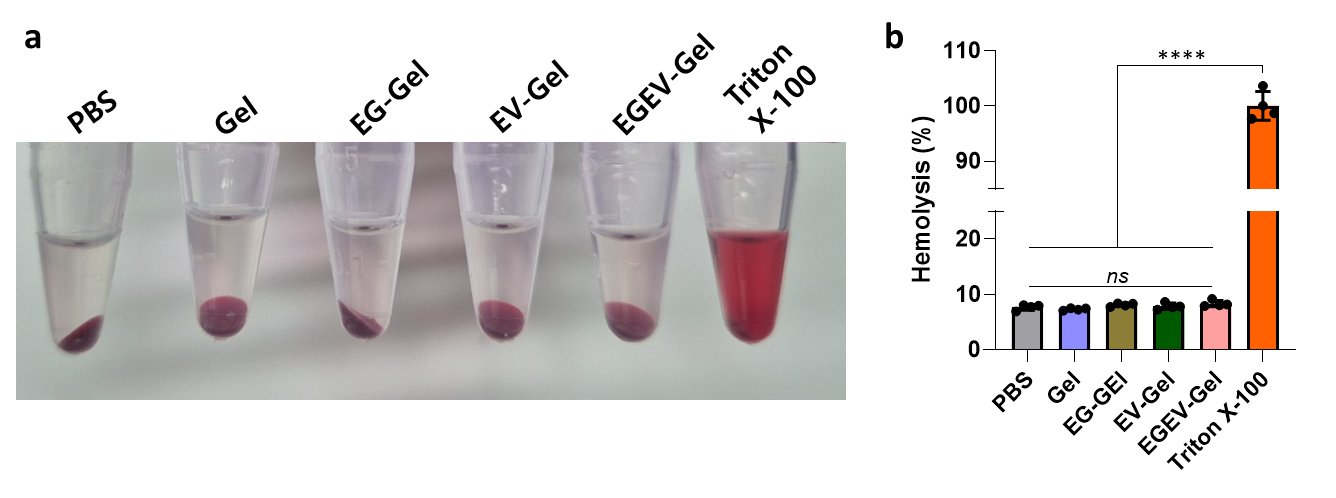


**Figure S15. Hemolysis evaluation of different hydrogel formulations.** (a) Representative images of red blood cell suspensions after incubation with PBS, Gel, EG-Gel, EV-Gel, and EGEV-Gel. (b) Quantitative analysis of hemolysis ratio for each group (n = 4). Statistical significances were calculated using the one-way ANOVA method (****p < 0.0001). Error bars represent the SD.


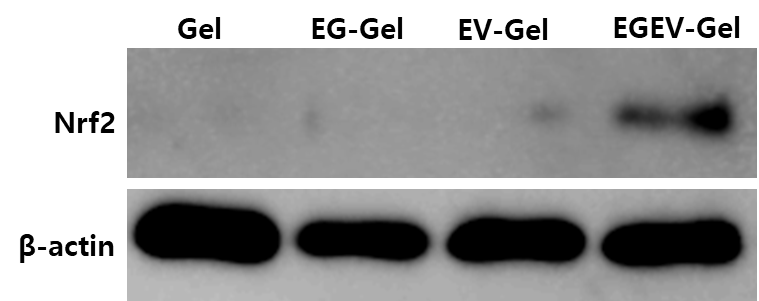


**Figure S16. Nrf2 expression in response to EGEV-Gel treatment *in vitro*.** Analysis of Nrf2 expression in activated BMDMs after treatment.


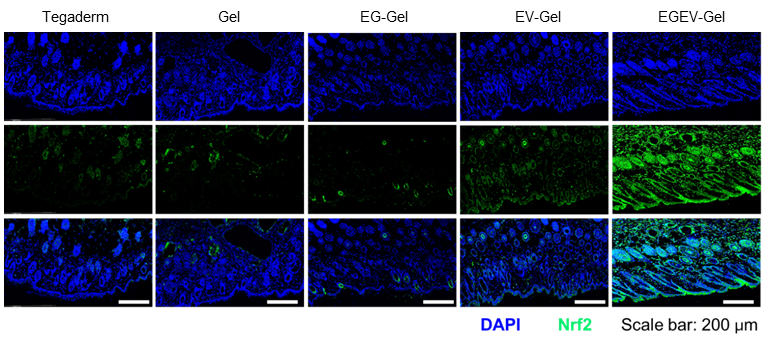


**Figure S17. In vivo Nrf2 expression after EGEV-Gel treatment**. Immunohistochemical staining of Nrf2 in wound tissues after 12-day treatment.

| Time (h) | EGCG release  (%; mean ± SD, n = 3) | hASC-EV release  (%; mean ± SD, n = 3) |
| --- | --- | --- |
| 0 | 0.00 ± 0.00 | 0.00 ± 0.00 |
| 1 | 79.60 ± 8.66 | 69.84 ± 9.59 |
| 6 | 84.58 ± 6.96 | 74.25 ± 9.44 |
| 12 | 86.32 ± 4.86 | 79.01 ± 8.11 |
| 24 | 87.46 ± 3.73 | 82.84 ± 7.08 |
| 48 | 88.18 ± 3.07 | 87.25 ± 6.62 |
| 72 | 88.43 ± 2.69 | 88.75 ± 6.69 |
| 96 | 88.43 ± 2.69 | 89.95 ± 6.47 |
| 144 | 88.43 ± 2.69 | 90.35 ± 6.47 |

**Table S1. Cumulative release profiles of EGCG and hASC-EVs from EGEV-Gel.**
